# Associations of Childhood Maltreatment With Suicidal Behavior Among Chinese Adolescents: Does It Differ Based on Gender and Biological Rhythm?

**DOI:** 10.3389/fpsyt.2022.885713

**Published:** 2022-07-08

**Authors:** Yang Xie, Huiqiong Xu, Baolin Wang, Xiaoyan Wu, Shuman Tao, Yuhui Wan, Fangbiao Tao

**Affiliations:** ^1^Department of Maternal, Child & Adolescent Health, School of Public Health, Anhui Medical University, Hefei, China; ^2^Anhui Provincial Key Laboratory of Population Health & Aristogenics, Anhui Medical University, Hefei, China; ^3^Moe Key Laboratory of Population Health Across Life Cycle, Anhui Medical University, Hefei, China; ^4^NHC Key Laboratory of Study on Abnormal Gametes and Reproductive Tract, Hefei, China; ^5^Department of Nephrology, The Second Hospital of Anhui Medical University, Hefei, China

**Keywords:** childhood maltreatment, adolescents, biological rhythm, suicidal planning, suicidal ideation, suicidal attempts

## Abstract

**Background:**

The impact of biological rhythm disorder (BRD) on the association of childhood maltreatment (CM) and suicidal behavior in adolescents remains unclear. CM increases the risk of suicidal ideation (SI), suicidal planning (SP), and suicidal attempts (SAs). There is less investigation on gender differences in CM's effects on suicidal behavior. It is unknown whether the impacts vary with different levels of BRD.

**Aims:**

To identify gender differences in CM's effects on suicidal behavior and to investigate these impacts at different levels of BRD.

**Method:**

The analysis is based on data from 7,986 adolescents recruited from three cities in China between October and December 2019. All participants, aged 14.7 ± 2 years, filled out standard questionnaires involving CM, BRD, and suicidal behavior.

**Results:**

A total of 22.9, 10.8, and 4.7% of the adolescents reported SI/SP/SAs in the past year. Girls are more likely to engage in SI and SP when exposed to the highest level of CM; boys are more likely to engage in SAs than girls. A significant relationship between moderate levels of CM and SI/SP/SAs was only observed in girls exposed to low BRD. Moderate CM is only significantly associated with SI in boys exposed to low BRD. The percentage of low-BRD adolescents who experienced high CM was 31.4%, whereas 58% of high-BRD adolescents experienced high CM in SI. Adolescents with high BRD were more likely to experience high levels of CM in SP and SAs.

**Conclusions:**

Adolescents at high risk of suicidal behavior in relation to CM should be targeted accordingly. Improving biological rhythm in adolescents who experience CM could help prevent them from engaging in suicidal behavior.

## Introduction

Suicidal behavior—including suicidal ideation (SI), suicidal planning (SP), and suicide attempts (SAs)—is a leading cause of death and disability worldwide and a serious global public health issue. More than 800,000 people die from suicide each year around the world, which indicates that one person dies by suicide every 40 seconds (1). Suicidal behavior is prevalent among adolescents across the globe; the prevalence of SI and SAs in American adolescents was 15.8 and 8.9%, respectively, in 2019 (2), and the rates of SI/SP/SAs among adolescents in China were 17.2, 13.6, and 7.4%, respectively (3). Suicidal behavior is associated with significant disability and psychosocial impairment among adolescents. They may seek information about suicide through prolonged exposure to the Internet and electronic media, which may lead to impaired clinical outcomes (4). Therefore, it is crucial to explore the potential risk factors for suicidal behavior. The underlying factors of suicidal behavior in adolescents are complex, and identifying risk factors for suicidal behavior will help advance effective prevention and early intervention. A substantial body of research has demonstrated the impact of childhood maltreatment (CM) on suicidal behavior. For instance, a prospective cohort study with 660 street adolescents aged 14 to 26 from Canada found that CM was a robust predictor of SA (5). A 3-year longitudinal study of adolescents aged 11.83 ± 2.42 in the US suggests that emotional maltreatment contributes uniquely to the prospective prediction of SI (6). A cross-sectional study with a representative sample of 21,019 high school students from China revealed that physical neglect, emotional neglect, physical abuse, emotional abuse, and sexual abuse were all associated with an increased risk of SI and SAs (7). A meta-analysis based on 79 individual studies found that younger participants with experiences of sexual abuse had higher rates of SAs, and younger age was linked more strongly with SI (8). Studies on multifactorial etiological models have shown that biological and social risk factors may be responsible for suicidal behavior by moderating the relationship between the latter and CM (9).

Meanwhile, we found that the reported rate of suicidal behavior among girls was higher than that of boys (10). Biological sex may be a vital factor in the examination of suicidal behavior. Evidence suggests that gender is a potential moderator that impacts the relationship between CM and suicidal behavior, and CM in girls may be more consistently linked to suicidal behavior than in boys (11, 12). However, previous studies show inconsistent results of the association between CM and suicidal behavior among boys. Martin found that self-reported sexual abuse was strongly and independently linked with suicidal thoughts, plans, and SAs only in boys (13). Other studies have found no sex differences (14). In response to these inconsistent findings, more research is necessary to verify whether the association between CM and suicidal behavior varies based on gender.

Biological rhythm disorder (BRD) is a new risk factor for suicidal behavior that is not fully understood. Research on biological rhythms and suicide dates back to 1959, when Dreyer first reported seasonal variations in suicide (15). Since then, researchers have found that vigorous circadian rhythm type appeared independently associated with SAs in individuals with bipolar disorder (16), and evening-type participants with major depressive disorder exhibited higher suicidal behavior (17). In addition, BRD promotes suicidal behavior in adolescents. A cross-sectional study with 1,667 adolescents (mean age: 14.8 ± 1.6 years old) in Hong Kong provides evidence that circadian issues are associated with SI (18). A substantial amount of literature has found a link between BRD and suicidal behavior, and current knowledge surrounding the association of BRD with suicidal behavior is predominantly derived from specific groups of people with various diseases and in Western countries. Although there are few international studies on the association between CM and BRD at present, existing research has reported that people who experience CM have an increased risk of circadian rhythm anomalies, which may be exhibited by abnormal cortisol secretion; CM may be associated with disturbances in cortisol secretion (19, 20). Despite a well-established link between CM and suicidal behavior, it is unknown whether the role of CM in this risk occurs through diverse and specific mechanisms (such as BRD).

Therefore, we hypothesized that gender and biological rhythm influence the association between CM and suicidal behavior. Our aims were to explore the impact of gender and biological rhythm on the relationship between CM and suicidal behavior through large-scale health survey data (*n* = 8,082) from typically developing adolescents in the Chinese cities of Shenzhen, Nanchang, and Shenyang.

## Methods

### Study Design and Participants

We derived the study population from a health survey. We chose three cities—Shenzhen, Nanchang, and Shenyang located in southern, central, and northern China, respectively—as our field sites for data collection based on economic development and geographic distribution. These cities are broadly representative of the well-developed economies in southern, central, and northern China, and average population in China in terms of economic level and demographic composition. These cities cooperated well with us, thereby facilitating data collection. In each city, we randomly selected 4 large middle schools, from which we recruited participants using stratified cluster sampling. First, in each school, we stratified students by grade. Thereafter, in different grades, we selected multiple classes and took all students in the classes as participants. In China, there are approximately 45 students in a class. Consequently, we chose 5 classes in each grade for the survey. Exclusion criteria included a family history of mental illness, the inability to complete the questionnaire, and unwillingness to participate in the investigation.

In total, 8,082 middle school students were given anonymous paper-based questionnaires in the classroom setting. There were 96 invalid questionnaires due to incomplete data because of adolescents' unwillingness to fill them out. The effective response rate was 98.8%. After excluding invalid questionnaires, a representative sample of 7,986 adolescents completed the standardized questionnaire, including 3,866 (48.4%) boys and 4,120 (51.6%) girls. Both the design and data collection were approved and reviewed by the Ethics Committee of Anhui Medical University. We gathered our survey data from October to December 2019, and all adolescents and their parents signed informed consent forms before the survey was administered.

### Measures

#### Sociodemographic Data

We collected sociodemographic data on each participant, including gender (boys or girls), age, registered residence (rural or urban), only child (yes or no), paternal and maternal education (<12 years or ≥12 years), and self-reported family economic status (high-income, middle-class, or low-income). In China, less than 12 years of schooling means that a person has not graduated from high school.

#### Childhood Maltreatment Experiences

We assessed CM with the Child Trauma Questionnaire (CTQ-SF), which is a standardized questionnaire for evaluating the history and frequency of CM in adolescents (21, 22). The CTQ-SF comprises 28 items (e.g., “My parents wish I had never been born,” “My family has said hurtful things to me,” “I have been hit hard enough to develop bruises,” and “I have worn dirty clothes”) and covers five categories, including emotional abuse, emotional neglect, physical abuse, physical neglect, and sexual abuse, three of which are calibration marks. Participants respond using a 5-point Likert scale (never true, rarely true, sometimes true, often true, and very often true). We classified the participants into tertiles of CTQ scores to identify individuals with severe exposure. The total scores ranged from 25 to 125, based on which we divided the participants into three groups according to the 67th and 33rd percentiles: a high level of CM, a moderate level of CM, and a low level of CM. The Cronbach's α coefficient of the CTQ-SF in this study was 0.867.

### Biological Rhythm Disorder

We used the Self-Rating of Biological Rhythm Disorder for Adolescents (SBRDA) (23) to assess BRD in the participants. The SBRDA comprises 29 items (e.g., “I need an alarm clock or someone else to wake me up in the morning,” “I skip breakfast one or two days a week,” and “I stay up late to use digital media”) that include four dimensions: sleep, digital media use, eating habits, and activity. Participants respond using a 5-point Likert scale (completely inconsistent, basically inconsistent, somewhat consistent, basically consistent, and completely consistent). We used the 90th percentile of total scores as the cut-off point to measure adolescents' BRD as the Chinese norm. The Cronbach's α coefficient of SBRDA in this study was 0.950.

### Suicidal Behavior

The Centers for Disease Control and Prevention used three questions to evaluate SI, SP, and SAs for the framework of the Youth Risk Behavior Survey System (YRBSS) (24):

“During the past year, have you ever considered suicide?”
“Have you made any plans about how to commit suicide in the last year?”
“Did you actually attempt suicide in the last year?”
All response options are coded as “yes” or “no.”

### Confounding Variables

We included multiple variables based on their potential association with suicidal behavior, including gender, registered residence (rural or urban), only child (yes or no), paternal and maternal education (<12 years or ≥12 years), self-reported family economic status (high-income, middle-class, or low-income), and city (Nanchang, Shenyang, or Shenzhen).

### Statistical Analysis

We performed all statistical analyses using SPSS version 23.0 (SPSS, Chicago, IL, USA). We conducted a chi-square test for categorical variables to compare the differences among behaviors. We employed binomial logistic regression models to assess the associations of the various behaviors with CM and adjusted for confounding variables. We fitted the adjusted logistic regression to identify determinants of suicidal behavior as the level of CM by BRD group and gender. We analyzed the interaction effects between CM with BRD and gender on suicidal behavior. We set statistical significance at *p* < 0.05 (two-tailed).

## Results

### Characteristics of the Participants

Of the 7,986 adolescents, the mean age and standard deviation were 14.7 ± 2 years. The prevalence of suicidal behavior is detailed in Table 1. In total, 22.9, 10.8, and 4.7% of the adolescents reported SI/SP/SAs. A total of 31.9% of the participants' registered residence was rural, and there were differences in SI and SP (*p* < 0.05). Participants who reported being only children showed a significantly higher rate of SAs (*p* < 0.05). Self-reported family economic status fell into the following groups: 12.9% were low-income, 66.2% were middle-class, and 20.9% were high-income. Compared with adolescents from high-income and middle-class families, those from low-income families had significantly more suicidal behavior (*p* < 0.001 for each, Table 1). Participants reported lower CM scores, a lower level of BRD, and fewer suicidal behavior (including SI, SP, and SAs) (*p* < 0.001). The rates of SI (25.7%), SP (12.4%) and SA (6.3%) among adolescents in Shenyang were significantly higher than those in the other two cities (Table 1). In addition, suicidal behavior revealed no statistically significant differences based on parents' education level.

**Table 1:** Descriptive statistics for variables.

**Variables**	**Total** ***N* = 7,986(%)**	**SI**		**SP**		**SAs**	
		***n*** **=** **1,825(%)**		***n*** **=** **859(%)**		***n*** **=** **378(%)**	
		***n* (%)**	***P* Value**	***n* (%)**	***P* Value**	***n* (%)**	***P* Value**
Gender			<0.001		<0.001		<0.001
Boy	3,866 (48.4)	686 (17.7)		315 (8.1)		118 (3.1)	
Girl	4,120 (51.6)	1,139 (27.6)		544 (13.2)		260 (6.3)	
Registered residence			<0.05		<0.05		0.572
Rural	2,547 (31.9)	529 (20.8)		247 (9.7)		115 (4.5)	
Urban	5,439 (68.1)	1,296 (23.8)		612 (11.3)		263 (4.8)	
City			<0.001		<0.05		<0.001
Nanchang	3,085 (38.6)	671 (21.8)		324 (10.5)		124 (4.0)	
Shenyang	2,563 (32.1)	659 (25.7)		317 (12.4)		161 (6.3)	
Shenzhen	2,338 (29.3)	495 (21.2)		218 (9.3)		93 (4.0)	
Only child			0.614		0.225		<0.05
Yes	2,769 (34.7)	642 (23.2)		314 (11.3)		156 (5.6)	
No	5,217 (65.3)	1,183 (22.7)		545 (10.4)		222 (4.3)	
Paternal education			0.095		0.535		0.832
<12 years	3,427 (42.9)	752 (21.9)		360 (10.5)		160 (4.7)	
≥ 12 years	4,559 (57.1)	1,073 (23.5)		499 (10.9)		218 (4.8)	
Maternal education			0.365		0.367		0.114
<12 years	3,891 (48.7)	872 (22.4)		406 (10.4)		169 (4.3)	
≥ 12 years	4,095 (51.3)	953 (23.3)		453 (11.1)		209 (5.1)	
Self-reported family economy			<0.001		<0.001		<0.001
Bad	1,030 (12.9)	335 (32.5)		162 (15.7)		73 (7.1)	
General	5,284 (66.2)	1,137 (21.5)		523 (9.9)		205 (3.9)	
Good	1,672 (20.9)	353 (21.1)		174 (10.4)		100 (6.0)	
CM			<0.001		<0.001		<0.001
Low	2,727 (34.1)	338 (12.4)		130 (4.8)		45 (1.7)	
Moderate	2,468 (30.9)	489 (19.8)		184 (7.5)		86 (3.5)	
High	2,791 (34.9)	998 (35.8)		545 (19.5)		247 (8.8)	
BRD			<0.001		<0.001		<0.001
Low	7,174 (89.8)	1 419 (19.8)		646 (9.0)		257 (3.6)	
High	812 (10.2)	406 (50.0)		213 (26.2)		121 (14.9)	

*SI, suicidal ideation; SP, suicidal planning; SAs, suicidal attempts; CM, childhood maltreatment; BRD, biological rhythm disorder. Statistical methods: Chi-square test*.

### Association of Suicidal Behaviors With CM

There was a gradient association of CM with suicidal behavior (Supplementary Table S1). Adolescents with high CM had a greater risk of SI/SP/SAs. After adjusting for gender, registered residence, self-reported family economic status, and city, the odds ratios (ORs) for adolescents with moderate CM vs. low CM in SI and SP were 1.8, 95% CIs: 1.6–2.1, *p* < 0.001 and 1.7, 95% CIs: 1.3–2.1, respectively. We noted a greater risk of SI and SP in adolescents with high CM (4.2, 95% CIs: 3.6–4.8, *p* < 0.001; 5.6, 95% CIs: 4.1–6.2, respectively) vs. low CM. After adjusting for gender, only child, self-reported family economic status, and city, compared to low CM, the ORs for adolescents with moderate and high levels of CM in SAs were 2.4 (1.6–3.4) and 6.5 (4.7–9.0), *p* < 0.001, respectively. After further adjusting for BRD, the gradient associations remained; a moderate level of CM in SI involved ORs of 1.8 (1.5–2.1), *p* < 0.001, and for high CM the ORs were 3.7 (3.2–4.3), *p* < 0.001. In SP, the ORs were 1.2 (1.3–2.0), *p* < 0.001, and for high CM, they were 4.5 (3.7–5.5), *p* < 0.001. In SAs, the ORs were 2.3 (1.6–3.3), *p* < 0.001, and 5.5 (3.9–7.6), *p* < 0.001.

### Interaction Effects Between CM With BRD and Gender on Suicidal Behavior

In the models, we tested the interaction effects between CM with BRD and gender on suicidal behavior. We observed significant interaction effects (see Supplementary Tables S2, S3). Compared to the reference group (low CM and low BRD), the interaction term of CM and BRD had an impact on suicidal behavior (*p* < 0.001). The higher the levels of CM and BRD, the greater the risk of suicidal behavior (see Supplementary Table S2). Compared to the reference group (low CM and girls), girls who experienced both moderate and high levels of CM had an increased risk of suicidal behavior. Boys with moderate CM had no significant risk of suicidal behavior compared to girls with moderate CM. Compared to girls who experienced high CM, boys who experienced high CM had a lower risk of suicidal behavior (see Supplementary Table S3).

### Association of Suicidal Behavior With CM in Boys and Girls

We further stratified the association between CM and suicidal behavior by gender and found that the higher risk of suicidal behavior in girls was upregulated with high CM (Table 2). Both moderate and high levels of CM in boys increased the risk of SI, whereas only high CM increased the risk in SP and SAs (Table 2).

**Table 2:** Number, % and OR of suicidal behaviors by level of CM in boys and girls.

**Variable**		**Yes** ***n* (%)**	**NO** ***n* (%)**	***P* Value**	**Model 1** **OR (95%CI)^**a**^**	**Model 2** **OR (95%CI)^**b**^**	**Model 3** **OR (95%CI)^**c**^**
SI	CM (tertile score)						
	*Boys*			<0.001			
	Low	113 (9.3)	1,098 (90.7)		1.0	1.0	1.0
	Moderate	171 (14.1)	1,042 (85.9)		1.6 (1.2~2.1)**	1.6 (1.2~2.0)*	1.5 (1.2~2.0)*
	High	402 (27.9)	1,040 (72.1)		3.8 (3.0~4.7)**	3.6 (2.9~4.5)**	3.3 (2.6~4.1)**
	*Girls*			<0.001			
	Low	225 (14.8)	1,291 (85.2)		1.0	1.0	1.0
	Moderate	318 (25.3)	937 (74.7)		1.9 (1.6~2.4)**	2.0 (1.7~2.4)**	2.0 (1.6~2.4)**
	High	596 (44.2)	753 (55.8)		4.5 (3.8~5.4)**	4.5 (3.8~5.4)**	4.0 (3.3~4.8)**
SP	CM (tertile score)						
	*Boys*			<0.001			
	Low	44 (3.6)	1,167 (96.4)		1.0	1.0	1.0
	Moderate	59 (4.9)	1,154 (95.1)		1.4 (0.9~2.0)	1.3 (0.9~2.0)	1.3 (0.9~1.9)
	High	212 (14.7)	1,230 (85.3)		4.6 (3.3~6.4)**	4.4 (3.1~6.1)**	4.0 (2.8~5.6)**
	*Girls*			<0.001			
	Low	86 (5.7)	1,430 (94.3)		1.0	1.0	1.0
	Moderate	125 (10.0)	1,130 (90.0)		1.8 (1.4~2.4)**	1.9 (1.4~2.5)**	1.8 (1.4~2.4)**
	High	333 (24.7)	1,016 (75.3)		5.5 (4.2~7.0)**	5.4 (4.2~7.0)**	4.8 (3.7~6.2)**
SAs	CM (tertile score)						
	*Boys*			<0.001			
	Low	12 (1.0)	1,199 (99.0)		1.0	1.0	1.0
	Moderate	21 (1.7)	1,192 (98.3)		1.8 (0.9~3.6)	1.8 (0.9~3.8)	1.7 (0.8~3.5)
	High	85 (5.9)	1,357 (94.1)		6.3 (3.4~11.5)**	6.3 (3.4~11.7)**	5.4 (2.9~10.0)**
	*Girls*			<0.001			
	Low	33 (2.2)	1,483 (97.8)		1.0	1.0	1.0
	Moderate	65 (5.2)	1,190 (94.8)		2.5 (1.6~3.8)**	2.6 (1.7~4.0)**	2.5 (1.7~3.9)**
	High	162 (12.0)	1,187 (88.0)		6.1 (4.2~9.0)**	6.4 (4.3~9.5)**	5.4 (3.7~8.1)**

*p < 0.05, **p < 0.001; SI, suicidal ideation; SP, suicidal planning; SAs, suicidal attempts; In SI and SP, a for crude model; ^b^Adjusted for registered residence, self-reported family economy, cities; ^c^Adjusted for registered residence, self-reported family economy, cities, and BRD. In SAs, a for crude model; b Adjusted for Only child, self-reported family economy, cities; c adjusted for Only child, self-reported family economy, cities, and BRD.

### Association of Suicidal Behavior With CM and BRD

Supplementary Table S4 shows stratified data analysis by BRD group in a crude model. According to Supplementary Table S4, there was a significant association of SI/SP/SAs with high CM in the low-BRD and high-BRD groups, but the relationship between high CM and suicidal behavior appeared stronger in these adolescents than in adolescents with low CM (all *p* < 0.05). As seen in Table 3, the results did not change significantly after further controlling for confounding factors. In SI and SP, adjusted for registered residence, self-reported family economic status, and city, we observed a significant association of high CM with suicidal behavior in low-BRD and high-BRD adolescents. In SAs, adjusted for only child, self-reported family economic status, and city, we witnessed the same positive association in high levels of CM and SA in the low-BRD and high-BRD groups. In both the crude and adjusted models, a moderate level of CM was positively correlated with SI/SP/SAs in the low-BRD group. In the high-BRD group, moderate CM was not associated with SI/SP/SAs. The percentage of low-BRD adolescents who experienced high CM was 31.4%, whereas 58% of high-BRD adolescents experienced high CM in SI. Adolescents with high BRD were more likely to experience high levels of CM in SP and SAs. Detailed values can also be found in Supplementary Table S4 and Table 3.

**Table 3:** Number, % and adjusted OR of suicidal behaviors by level of CM and BR.

**CM (tertile score)**	**LBRD** ^**a**^		**HBRD** ^**b**^	
	***n* (%)**	**OR (95%CI)**	***n* (%)**	**OR (95%CI)**
SI				
Low	280 (10.9)	1.0	58 (37.9)	1.0
Moderate	406 (17.9)	1.8 (1.5~2.1)**	83 (41.1)	1.2 (0.8~1.8)
High	733 (31.4)	3.7 (3.1~4.3)**	265 (58.0)	2.3 (1.6~3.4)**
SP				
Low	109 (4.2)	1.0	21 (13.7)	1.0
Moderate	149 (6.6)	1.6 (1.2~2.0)**	35 (17.3)	1.4 (0.8~2.5)
High	388 (16.6)	4.4 (3.5~5.5)**	157 (34.4)	3.4 (2.0~5.6)**
SAs				
Low	31 (1.2)	1.0	14 (9.2)	1.0
Moderate	70 (3.1)	2.7 (1.8~4.2)**	16 (7.9)	0.9 (0.4~2.0)
High	156 (6.7)	6.0 (4.1~9.0)**	91 (19.9)	2.7 (1.5~5.0)*

*p < 0.05, **p < 0.001; SI, suicidal ideation; SP, suicidal planning; SAs, suicidal attempts; LBRD, low biological rhythm disorder; HBRD, high biological rhythm disorder. In SI and SP, ^a^Adjusted for registered residence, self-reported family economy, cities; In SAs, ^b^Adjusted for Only child, self-reported family economy, cities.

## Discussion

We drew upon data from a large school-based survey in China to illuminate the association of suicidal behavior with CM in girls and boys and its variation with the levels of BRD. To our knowledge, this is the first study to explore the association between CM and BRD with suicidal behavior in adolescents. A key and novel contribution of this study is that it illustrates the influence of BRD on the relationship between CM and SI, SP, and SAs. Our results indicate that both moderate and high CM levels were associated with suicidal behavior in adolescents with low BRD, whereas only high CM was associated with suicidal behavior in adolescents with high BRD. Furthermore, adolescents with high BRD were more likely to experience high CM, and adolescents with low BRD were less likely to experience CM. According to the stratification analyses, our data clarify that both male and female adolescents with high CM may have a higher risk of suicidal behavior (SI/SP/SAs). Compared to boys, girls are generally more likely to engage in SI and SP when exposed to the highest level of CM; however, boys are more likely to engage in SAs than girls. Moreover, CM, as a critical lifetime event, had a stronger effect on SI/SP/SAs among adolescents with low BRD than among adolescents with high BRD. We only observed a significant relationship between moderate levels of CM and SI/SP/SAs in girls exposed to low BRD. Moderate CM was only significantly associated with SI in boys exposed to low BRD.

Previous research has found that individuals with high CM have a greater risk of engaging in suicidal behavior, which is consistent with our results (25, 26). Data from a total of 10,148 US adolescents revealed that those who were exposed to either physical or sexual abuse had an increased risk of reporting SI/SP/SAs (27). A cross-sectional survey in South India among 13- to 14-year-old girls found that SI was independently linked to sexual abuse and low parental emotional support (28). Emotional neglect, physical abuse, and physical neglect increased the risk of SAs among adolescents and young adults (29). Our results of vulnerability among girls, especially those with the highest CM, are similar to those in former studies. There is a stronger positive relationship between physical abuse and SI among girls (30). Young girls with more adverse childhood experiences have a higher risk of SI and SP (31). Gallo asserted that girls are vulnerable to low self-esteem due to childhood abuse (32) and are therefore prone to mental health problems that can lead to SI and SP. Our findings suggest that boys who experience CM are more likely to engage in SAs, although the ORs were similar to those of girls, possibly because adolescent boys are more likely to engage in impulsive behavior (33). A considerable proportion of SAs are made on impulse (34).

Overall, these findings offer cogent evidence of the relationship between CM and SI/SP/SAs across BRD groups in adolescents. Our study has advanced the literature by making three unique contributions. First, with a total of 7,986 adolescents, this is a representative large sample from three regions in China that exhibits ORs for the association between CM and SI/SP/SAs. Second, this study is the first, to our knowledge, to offer evidence of the strong link between CM and suicidal behavior among boys and girls. A third important contribution is the identification of a new perspective that moderates the association between CM and suicidal behavior in adolescents. In particular, we found that adolescents with high BRD were more likely to experience high CM, and CM was associated with suicidal behavior in girls who had high BRD and low BRD. This outcome has vital implications; in that, it highlights an urgent need to improve BRD as part of suicide behavior prevention and intervention strategies among adolescents who have experienced CM.

## Limitations

There are several limitations to our study. First, the study had a cross-sectional design and we could not determine the priority of CM, BR, and suicidal behavior. Follow-up studies should be carried out to strengthen the validity of causal associations. Second, we did not analyze CM by category, but instead used comprehensive indicators of CM to explore the risk of suicide in different BRD groups. It may not be possible to tell which specific types of childhood abuse have a greater effect on suicidal behavior. Meanwhile, we used self-reported data, which has certain information bias. Third, the risk factors for suicidal behavior are complex. Although we fully considered gender and biological rhythm, more comprehensive attention should be given to psychosocial factors, such as affective temperament (35), and further studies should be conducted on the impact of the interaction between psychosocial factors and biological rhythm on suicidal behavior. Despite these limitations, the large sample size deepens our understanding of the impact of gender and biological rhythm on the relationship between CM and suicidal behavior.

## Conclusion

As a public health problem, suicidal behavior in adolescents merits discussion. Our results highlight the need to incorporate amelioration of CM's influence and the improvement of biological rhythm into suicidal behavior prevention strategies, and in efforts to raise public awareness. These findings have implications for the multifactorial etiological model in which biological and social risk factors, such gender and biological rhythm, can moderate the relationship between CM and suicidal behavior. Thus, both male and female adolescents with BRD should be considered in the implementation of effective intervention programs related to suicide. It is crucial that public health policy in China encompasses planned intervention programs to reduce the rate of suicidal behavior.

## Data Availability Statement

The raw data supporting the conclusions of this article will be made available by the authors, without undue reservation.

## Ethics Statement

The studies involving human participants were reviewed and approved by the Ethics Committee of Anhui Medical University. Written informed consent to participate in this study was provided by the participants' legal guardian/next of kin.

## Author Contributions

YX, HX, and BW: conceptualization and formal analysis. YX, YW, and FT: writing original draft. YX, XW, YW, ST, and FT: investigation. YX and YW: methodology. FT: supervision, funding acquisition, and writing review and editing. All authors checked interpreted results and approved the final version.

## Funding

The study was funded by National Natural Science Foundation of China (Grant number: 82073578).

## Conflict of Interest

The authors declare that the research was conducted in the absence of any commercial or financial relationships that could be construed as a potential conflict of interest.

## Publisher's Note

All claims expressed in this article are solely those of the authors and do not necessarily represent those of their affiliated organizations, or those of the publisher, the editors and the reviewers. Any product that may be evaluated in this article, or claim that may be made by its manufacturer, is not guaranteed or endorsed by the publisher.
