# Supplementary material for: Associations of Childhood Maltreatment With Suicidal Behavior Among Chinese Adolescents: Does It Differ Based on Gender and Biological Rhythm?
Source: Front Psychiatry. 2022 Jul 8;13:885713. doi: 10.3389/fpsyt.2022.885713 (PMC9309254; doi:10.3389/fpsyt.2022.885713)
Supplement: Supplementary file 1 [file Table_1.docx]

Additional material

| Tab. DS1. Association of suicidal behaviors with CM | | | | |
| --- | --- | --- | --- | --- |
| Variable | | Adjusted OR (95%CI)^a^ |  | Adjusted OR (95%CI)^b^ |
| SI | CM (tertile score) |  |  |  |
|  | Low | 1.0 |  | 1.0 |
|  | Moderate | 1.8 (1.6 ~ 2.1)** |  | 1.8 (1.5 ~ 2.1)** |
|  | High | 4.2 (3.6 ~ 4.8)** |  | 3.7 (3.2 ~ 4.3)** |
| SP | CM (tertile score) |  |  |  |
|  | Low | 1.0 |  | 1.0 |
|  | Moderate | 1.7 (1.3 ~ 2.1)** |  | 1.6 (1.3 ~ 2.0)** |
|  | High | 5.6 (4.1 ~ 6.2)** |  | 4.5 (3.7 ~ 5.5)** |
| SAs | CM (tertile score) |  |  |  |
|  | Low | 1.0 |  | 1.0 |
|  | Moderate | 2.4 (1.6 ~ 3.4)** |  | 2.3 (1.6 ~ 3.3)** |
|  | High | 6.5 (4.7 ~ 9.0)** |  | 5.5 (3.9 ~ 7.6)** |

Note. **p* < .05, ***p* < .001; SI is Suicidal ideation; SP is Suicidal planning; SAs is Suicidal attempts; In SI and SP, a Adjusted for gender, registered residence, self-reported family economy, cities; b Adjusted for gender, registered residence, self-reported family economy, cities, and BRD. In SAs, a Adjusted for gender, Only child, self-reported family economy, cities; b adjusted for gender, Only child, self-reported family economy, cities, and BRD.

| Tab. DS2. Association of CM, BRD, and suicidal behaviors | | | | |
| --- | --- | --- | --- | --- |
| Variable | | Crude OR (95%CI) |  | Adjusted OR (95%CI)^a^ |
| SI | CM×BRD |  |  |  |
|  | LCM×LBRD | 1.0 |  | 1.0 |
|  | MCM×LBRD | 1.8 (1.5 ~ 2.1)** |  | 1.9 (1.6 ~ 2.2)** |
|  | HCM×LBRD | 3.8 (3.2 ~ 4.4)** |  | 4.0 (3.4 ~ 4.7)** |
|  | LCM×HBRD | 5.0 (3.5 ~ 7.1)** |  | 4.5 (3.2 ~ 6.5)** |
|  | MCM×HBRD | 5.7 (4.2 ~ 7.8)** |  | 5.7 (4.2 ~ 7.8)** |
|  | HCM×HBRD | 11.3 (9.0 ~ 14.1)** |  | 10.9 (8.7 ~ 13.7)** |
| SP | CM×BRD |  |  |  |
|  | LCM×LBRD | 1.0 |  | 1.0 |
|  | MCM×LBRD | 1.6 (1.2 ~ 2.1)** |  | 1.6 (1.3 ~ 2.1)** |
|  | HCM×LBRD | 4.5 (3.6 ~ 5.6)** |  | 4.7 (3.8 ~ 5.9)** |
|  | LCM×HBRD | 3.6 (2.2 ~ 5.9)** |  | 3.2 (2.0 ~ 5.3)** |
|  | MCM×HBRD | 4.7 (3.1 ~ 7.2)** |  | 4.7 (3.1 ~ 7.1)** |
|  | HCM×HBRD | 11.8 (9.0 ~ 15.5)** |  | 11.3 (8.5 ~ 14.9)** |
| SAs | CM×BRD |  |  |  |
|  | LCM×LBRD | 1.0 |  | 1.0 |
|  | MCM×LBRD | 2.6 (1.7 ~ 4.0)** |  | 2.9 (1.9 ~ 4.4)** |
|  | HCM×LBRD | 5.9 (4.0 ~ 8.7)** |  | 6.6 (4.5 ~ 9.9)** |
|  | LCM×HBRD | 8.3 (4.3 ~ 15.9)** |  | 6.8 (3.5 ~ 13.3)** |
|  | MCM×HBRD | 7.1 (3.8 ~ 13.1)** |  | 6.9 (3.7 ~ 12.8)** |
|  | HCM×HBRD | 20.4 (13.4 ~ 31.1)** |  | 19.8 (12.9 ~ 30.5)** |

Note. **p* < .05, ***p* < .001; SI is suicidal ideation; SP is suicidal planning; SAs is suicidal attempts; In SI and SP, a Adjusted for gender, registered residence, self-reported family economy, cities. In SAs, a Adjusted for gender, Only child, self-reported family economy, cities; LBRD is low biological rhythm disorder; HBRD is high biological rhythm disorder; LCM is low childhood maltreatment, MCM is moderate childhood maltreatment, HCM is high childhood maltreatment.

| Tab. DS3. Association of CM, gender, and suicidal behaviors | | | | |
| --- | --- | --- | --- | --- |
| Variable | | Crude OR (95%CI) |  | Adjusted OR (95%CI)^a^ |
| SI | CM×gender |  |  |  |
|  | LCM×girls | 1.0 |  | 1.0 |
|  | MCM×girls | 1.9 ( 1.6~ 2.3)** |  | 2.0 (1.6 ~ 2.4)** |
|  | HCM×girls | 4.5 ( 3.8~ 5.4)** |  | 4.5 (3.7 ~ 5.4)** |
|  | LCM×boys | 0.6 (0.5 ~ 0.8)** |  | 0.6 (0.5 ~ 0.7)** |
|  | MCM×boys | 0.9 (0.8 ~ 1.2) |  | 0.9 (0.7 ~ 1.1) |
|  | HCM×boys | 2.2 (1.8 ~ 2.7)** |  | 2.1 (1.8 ~ 2.5)** |
| SP | CM×gender |  |  |  |
|  | LCM×girls | 1.0 |  | 1.0 |
|  | MCM×girls | 1.8 (1.4 ~ 2.4)** |  | 1.9 (1.4 ~ 2.5)** |
|  | HCM×girls | 5.5 (4.2 ~ 7.0)** |  | 5.4 (4.2 ~ 6.9)** |
|  | LCM×boys | 0.6 (0.4 ~ 0.9)* |  | 0.6 (0.4 ~ 0.9)* |
|  | MCM×boys | 0.9 (0.6 ~ 1.2) |  | 0.8 (0.6 ~ 1.2) |
|  | HCM×boys | 2.9 (2.2 ~ 3.7)** |  | 2.7 (2.1 ~ 3.6)** |
| SAs | CM×gender |  |  |  |
|  | LCM×girls | 1.0 |  | 1.0 |
|  | MCM×girls | 2.5 (1.6 ~ 3.8)** |  | 1.9 (1.4 ~ 2.5)** |
|  | HCM×girls | 6.1 (4.2 ~ 9.0)** |  | 5.4 (4.2 ~ 7.0)** |
|  | LCM×boys | 0.5 (0.2 ~ 0.9)* |  | 0.6 (0.4 ~ 0.9)* |
|  | MCM×boys | 0.8 (0.5 ~ 1.4) |  | 0.8 (0.6 ~ 1.2) |
|  | HCM×boys | 2.8 (1.9 ~ 4.2)** |  | 2.7 (2.1 ~ 3.6)** |

Note. **p* < .05, ***p* < .001; SI is suicidal ideation; SP is suicidal planning; SAs is suicidal attempts; In SI and SP, a Adjusted for registered residence, self-reported family economy, cities. In SAs, a Adjusted for Only child, self-reported family economy, cities; LCM is low childhood maltreatment, MCM is moderate childhood maltreatment, HCM is high childhood maltreatment.

| Tab. DS4. Number, % and OR of suicidal behaviors by level of CM and BRD | | | | | |
| --- | --- | --- | --- | --- | --- |
| CM (tertile score) | LBRD | |  | HBRD | |
|  | n (%) | OR (95%CI) |  | n (%) | OR (95%CI) |
| SI |  |  |  |  |  |
| Low | 280 (10.9) | 1.0 |  | 58 (37.9) | 1.0 |
| Moderate | 406 (17.9) | 1.8 (1.5 ~ 2.1)** |  | 83 (41.1) | 1.1 (0.7 ~ 1.8) |
| High | 733 (31.4) | 3.8 (3.2 ~ 4.4)** |  | 265 (58.0) | 2.3 (1.6 ~ 3.3)** |
| SP |  |  |  |  |  |
| Low | 109 (4.2) | 1.0 |  | 21 (13.7) | 1.0 |
| Moderate | 149 (6.6) | 1.6 (1.2 ~ 2.1)** |  | 35 (17.3) | 1.3 (0.7 ~ 2.4) |
| High | 388 (16.6) | 4.5 (3.6 ~ 5.6)** |  | 157 (34.4) | 3.3 (2.0 ~ 5.4)** |
| SAs |  |  |  |  |  |
| Low | 31 (1.2) | 1.0 |  | 14 (9.2) | 1.0 |
| Moderate | 70 (3.1) | 2.6 (1.7 ~ 4.0)** |  | 16 (7.9) | 0.9 (0.4 ~ 1.8) |
| High | 156 (6.7) | 5.9 (4.0 ~ 8.7)** |  | 91 (19.9) | 2.5 (1.4 ~ 4.5)* |

Note. **p* < .05, ***p* < .001; Crude model. Note. **p* < .05, ***p* < .001; SI is suicidal ideation; SP is suicidal planning; SAs is suicidal attempts; In SI and SP; LBRD is low biological rhythm disorder; HBRD is high biological rhythm disorder.
